# Human Factors Evaluation of HeartMate 3 Left Ventricular Assist Device Peripherals: An Eye Tracking Supported Simulation Study

**DOI:** 10.1007/s10916-023-01950-3

**Published:** 2023-05-03

**Authors:** Gregor Widhalm, Theodor Abart, Moritz Noeske, Lisa Kumer, Katharina Ebenberger, Clemens Atteneder, Angelika Berger, Günther Laufer, Dominik Wiedemann, Daniel Zimpfer, Heinrich Schima, Michael Wagner, Thomas Schlöglhofer

**Affiliations:** 1 Department of Cardiac Surgery, Medical University of Vienna, Vienna, Austria; 2 Division of Neonatology, Pediatric Intensive Care and Neuropediatrics, Department of Pediatrics, Comprehensive Center for Pediatrics, Medical University of Vienna, Vienna, Austria; 3 Ludwig Boltzmann Institute for Cardiovascular Research, Vienna, Austria; 4 Center for Medical Physics and Biomedical Engineering, Medical University of Vienna, Vienna, Austria

**Keywords:** Left ventricular assist device, Eye tracking, Human factors, Usability, Peripherals, Simulation

## Abstract

**Background:**

Despite recent design improvements, human factors issues continue to challenge left ventricular assist device (LVAD) therapy. The aim of this study was to evaluate user experience of former non-HeartMate 3 (HM3) LVAD patients post heart transplantation (HTX) and laypersons (LP) with HM3 LVAD peripherals in simulated everyday and emergency scenarios.

**Methods:**

This single center cohort study included untrained HTX and LP. Seven scenarios, including battery exchanges (without alarm, advisory alarm, dim light, consolidated bag), change of power supply, driveline dis-/reconnection and controller exchange were simulated. Subjects’ gaze behavior was recorded using eye tracking technology. Success rate, pump-off-time, duration to success (DTS), percental fixation duration per areas of interest and post-scenario-survey results were defined as outcome measures.

**Results:**

Thirty subjects completed 210 scenarios, initially solving 82.4% (HTX vs. LP, p = 1.00). Changing power supply revealed highest complexity (DTS = 251 ± 93s, p = 0.76): 26.7% succeeded at first attempt (p = 0.68), 56.7% at second attempt, with significantly more LP failing (p = 0.04), resulting in 10 hazards from driveline disconnections (pump-off-time 2-118s, p = 0.25). Comparison on initial success showed differences in fixation durations for seven areas of interest (p < 0.037). Decreasing DTS during battery exchanges (p < 0.001) indicate high learnability. Exchanging batteries within the bag took longer (median DTS = 75.0 (IQR = 45.0)s, p = 0.09), especially in elderly subjects (r = 0.61, p < 0.001). Subjects with less initial success were more afraid of making mistakes (p = 0.048).

**Conclusion:**

This eye tracking based human factors study provided insights into user experiences in handling HM3 peripherals. It highlights unintuitive and hazardous characteristics, providing guidance for future user-centered design of LVAD wearables.

**Supplementary Information:**

The online version contains supplementary material available at 10.1007/s10916-023-01950-3.

## Introduction

Due to the mismatch between available donor organs and end stage heart failure patients [1], left ventricular assist device (LVAD) therapy represents a valuable treatment option, whether as bridge to heart transplantation, bridge to candidacy or destination therapy [2]. Continued improvements in pump design and technology increased LVAD patient survival to 83.0% one year and 74.7% two years post-implantation [2]. The currently available HeartMate 3 (HM3) LVAD (Abbott, Chicago, Illinois, USA) system shows excellent clinical outcomes [3, 4]. However, this therapy consists not only of the implanted pump but also of external, wearable components connected via a percutaneous driveline that provide power supply and monitor pump performance [5]. Although research has focused extensively on pump technology, adverse events, and clinical outcomes [2–4], less attention has been paid to the wearable components of LVAD devices [6–12]. These wearables show a variety of usability and user experience issues, affecting not only patients’ quality of life, but also patient safety [7–11]. LVAD device malfunction more frequently affect peripheral devices including controller, battery and clips, or driveline failure compared to failing pump components [13]. As previously reported, one-third of trained LVAD patients feel unprepared for emergency situations [12], indicating limited intuitiveness and usability. Intuitive, self-explanatory and convenient handling is an essential requirement for medical devices, especially for highly critical life support systems as LVADs [10].

Eye tracking (ET) technology systems are a valuable tool for quantitative assessment of human factors and user experience when handling and designing medical devices [14, 15]. They have demonstrated their potential for quantitatively and objectively assessing patients’ task performance [16], understanding of participants’ gaze behavior [17], improving user-centered medical device design [18, 19], and evaluating the effectiveness of training [20, 21]. ET analysis allows the calculation of areas of interest (AOI) specific metrics, such as mean fixation duration or dwell time per AOI [22], which can be used to identify components of the device under test that are of particular focus.

The aim of this study was to evaluate the intuitive handling and user experience with HM3 LVAD peripherals of former non-HM3 LVAD patients who underwent heart transplantation (HTX) and laypersons (LP) without previous training in ET supported simulations of everyday and emergency situations. The primary outcome was the difference in success rate and duration to success (DTS) per scenario between untrained LP and HTX patients. Secondary outcomes included the difference in percental fixation duration per AOI between initially successful (iSUC) and initially unsuccessful (iUNS) subjects per scenario, correlations between demographics and scenario performing measures, and user experience based on the post-scenario survey.

## Materials and Methods

This cross-sectional, single center cohort simulation study was conducted between February 2021 and December 2022. Seven predefined scenarios were simulated in a dedicated simulation center (closed room), implicating constant conditions for all subjects. The study protocol was approved by the Institutional Review Board (identification number: EK2034/2021), and all subjects provided written informed consent.

### Study Population

Former non-HM3 LVAD patients after HTX and LP without prior LVAD experience, aged > 18years, were included in this study. A 1:1 case matching was performed based on age and gender. HTX patients with devices other than isolated LVAD, less than 6 months LVAD support, more than 10 years since HTX, and subjects with language barriers were not included.

### Simulation Setting

Simulations were recorded using a 4-perspective room recording system (SimStation Pro, SIMStation GmbH, Vienna, Austria). Study subjects were equipped with ET glasses (Tobii Pro Glasses 2, Tobii AB, Stockholm, Sweden) for retrospective analysis of subjects’ gaze behavior. Seven pre-defined everyday and emergency scenarios that LVAD patients may face during the course of their therapy were simulated [12]:

1. Battery exchange without alarm.
2. Change of power supply: Battery operation to alternating current (AC) and reconnection of batteries.
3. Battery exchange as reaction to advisory alarm.
4. Driveline dis- and reconnection.
5. Emergency controller exchange as reaction to hazard alarm.
6. Battery exchange in dim light.
7. Battery exchange within consolidated bag.

The untrained subjects were introduced to the study procedure and the ET technology before the simulations. The fully functional components required for each scenario were placed on a table in front of the subjects, who received a brief introduction prior to each scenario (see Supplementary File 1). All scenarios were time capped at 3 min, except for the emergency controller exchange (5 min). If participants were not able to solve the task, the subjects were provided with an explanation followed by a second attempt.

### Date Collection and Processing

Prior to the simulations, baseline demographics, highest completed education, comorbidities, clinical frailty score [23] and EQ-5D-5 L questionnaire [24] were assessed. In addition, the dimensions of the subjects’ dominant hand were determined [25, 26].

Scenario performance was based on task duration per attempt and DTS, number and types of errors, success rate, number of attempts, and the pump-off time. ET was recorded at a sampling frequency of 50 Hz, based on corneal reflection dark pupil technology [27] and an one-point calibration was performed prior to each scenario. Post processing of the ET data was performed using Tobii Pro Lab version 1.207 (Tobii AB, Stockholm, Sweden). Recordings with a gaze sample percentage < 75% and recordings originating from the scenarios in dim light or the battery exchange within the bag were excluded from analysis. Areas of interest (AOIs) were defined for both, relevant individual HM3 components and connectors (see Supplementary File 2). Due to the highly dynamic nature of subjects’ movements during the scenarios, the Tobii I-VT (Attention) filter (velocity threshold of 100 degrees/second) was used. Single fixations were manually mapped to the corresponding area of interests on a snapshot of the situation. To correct for different time of interest (introduction and task) durations per subject, the percental fixation duration per AOI was computed. Heat maps were generated to allow visual interpretation of differences in gaze behavior. Pupil diameters were further processed using Matlab R2021b (The MathWorks Inc., Natick, MA, USA) to gain mean diameters per time of interest.

Following the simulated scenarios, subjects completed an 18-item Likert-Scale survey focusing user experience and device handling.

### Statistical Analysis

SPSS for Windows Release 28.0.0 (IBM, NY, USA) was used for statistical analyses. Variables were tested for normal distribution using the Shapiro-Wilk-Test. Descriptive statistics are presented as mean ± standard deviation for normally distributed data and as median (interquartile range (IQR)) for non-normally distributed continuous variables, while categorical variables are provided as number (percentage) or median percentage [range] if the number of cases was ≤ 3. Baseline characteristics, scenario performances, survey results and ET results (n ≥ 3) were compared based on the cohort (HTX vs. LP) and initial success (iSUC vs. iUNS) using the Fisher’s exact test for categorical variables, and depending on the normal distribution, the unpaired t-test or Mann-Whitney U test for independent continuous variables. Pearson and Spearman correlations were applied to test for relationships between subjects’ task performance parameters and demographic information. Statistical significance was set to p < 0.05.

## Results

### Patient Characteristics

The study included 30 subjects (15 HTX vs. 15 LP), 20.0% female, aged 63.5 (10.0) years without significant differences between the cohorts in age (p = 0.84), gender (p = 1.00) or body mass index (p = 0.12), who completed 210 scenarios. HTX subjects were former HeartMate II (Abbott, Chicago, Illinois, USA) (n = 2, 13.3%) or HVAD (Medtronic plc, Minnesota, USA) (n = 13, 86.7%) patients. Baseline demographics and socioeconomic status of the overall study population and stratified by the cohorts are summarized in Table 1.

**Table 1:** Baseline demographics, comorbidities and highest completed education for the overall study population and stratified by the two cohorts: former non-HM3 LVAD patients post heart transplantation and laypersons. * p-value comparing HTX vs. LP cohort. HTX: heart transplantation; LVAD: left ventricular assist device; LP: laypersons; NYHA: New York Heart Association; CMP: cardiomyopathy, VAS: visual analogue scale, IQR: interquartile range, STD: standard deviation

Variable Median (IQR) or mean ± STD or n (%)	Full cohort (n = 30)	HTX (n = 15)	Laypersons (n = 15)	p-value*
**Baseline Demographics**				
Age in years	63.5 (10.0)	64 (11.0)	63 (10.0)	0.84
Gender				1.00
Male	24 (80.0%)	12 (80.0%)	12 (80.0%)	
Female	6 (20.0%)	3 (20.0%)	3 (20.0%)	
Height in cm	176 (10)	172 (12)	176 (9)	0.74
Weight in kg	75.5 (22.3)	74.0 (16.0)	76.0 (26.0)	0.38
Body mass index in kg/m^2^	25.7 (5.5)	25.0 (4.3)	28.3 (7.0)	0.12
Dominant arm				1.00
Right	28 (93.3%)	15 (100%)	13 (86.7%)	
Left	1 (3.3%)	0 (0.0%)	1 (6.7%)	
Both	1 (3.3%)	0 (0.0%)	1 (6.7%)	
Hand width in cm	2.6 ± 0.3	2.6 ± 0.3	2.6 ± 0.3	0.70
Hand length in cm	18.5 (2.0)	19.0 (1.0)	18.0 (1.5)	0.25
Thumb-to-middle-finger-span in cm	17.3 ± 1.5	16.9 ± 1.5	17.7 ± 1.4	0.16
Thumb diameter in cm	1.81 ± 0.2	1.8 ± 0.2	1.8 ± 0.2	0.61
History of stroke				1.00
Positive anamnesis	1 (3.3%)	0 (0.0%)	1 (6.7%)	
Negative anamnesis	29 (96.7%)	15 (100%)	14 (93.3%)	
Medical condition affecting HFE task completion.				
Arthritis	1 (3.3%)	0 (0.0%)	1 (6.7%)	1.00
Neuropathy	4 (13.3%)	1 (6.7%)	3 (20.0%)	0.60
Hearing impairment	6 (20.0%)	3 (20.0%)	3 (20.0%)	1.00
Mobility impairment	7 (23.3%)	3 (20.0%)	4 (26.7%)	1.00
Vision impairment	25 (83.3%)	12 (80.0%)	13 (86.7%)	1.00
Highest completed education				
Secondary school	3 (10.0%)	2 (13.3%)	1 (6.7%)	0.19
Vocational school	7 (23.3%)	6 (40.0%)	1 (6.7%)	
Intermediate vocational school	4 (13.3%)	2 (13.3%)	2 (13.3%)	
A-levels	7 (23.3%)	2 (13.3%)	5 (33.3%)	
University degree	9 (30.0%)	3 (20.1%)	6 (40.0%)	
Clinical frailty score				0.61
CFS 1	1 (3.3%)	1 (6.7%)	0 (0.0%)	
CFS 2	21 (70.0%)	9 (60.0%)	12 (80.0%)	
CFS 3	5 (16.7%)	3 (20.0%)	2 (13.3%)	
CFS 4	2 (6.7%)	1 (6.7%)	1 (6.7%)	
CFS 7	1 (3.3%)	1 (6.6%)	0 (0.0%)	
EQ-5D-5 L				
VAS in %	85.0 (16.0)	80.0 (15.0)	90.0 (15.0)	0.08
Index	0.98 (0.10)	0.93 (0.19)	1.00 (0.06)	0.08
NYHA stage				
NYHA 1	-	11 (73.3%)	-	-
NYHA 2	-	3 (20.0%)	-	-
NYHA 3	-	1 (6.7%)	-	-
NYHA 4	-	0 (0.0%)	-	-
LVAD type before HTX				
HeartMate II	-	2 (13.3%)	-	-
HVAD	-	13 (86.7%)	-	-
Days on LVAD support before HTX	-	617 (734)	-	-
Days since HTX	-	2190 ± 996	-	-
Underlying Disease				
Ischemic CMP	-	14 (93.3%)		-
Restrictive CMP	-	1 (6.7%)	- -	-

### Scenario Performances

**Fig. 1:**
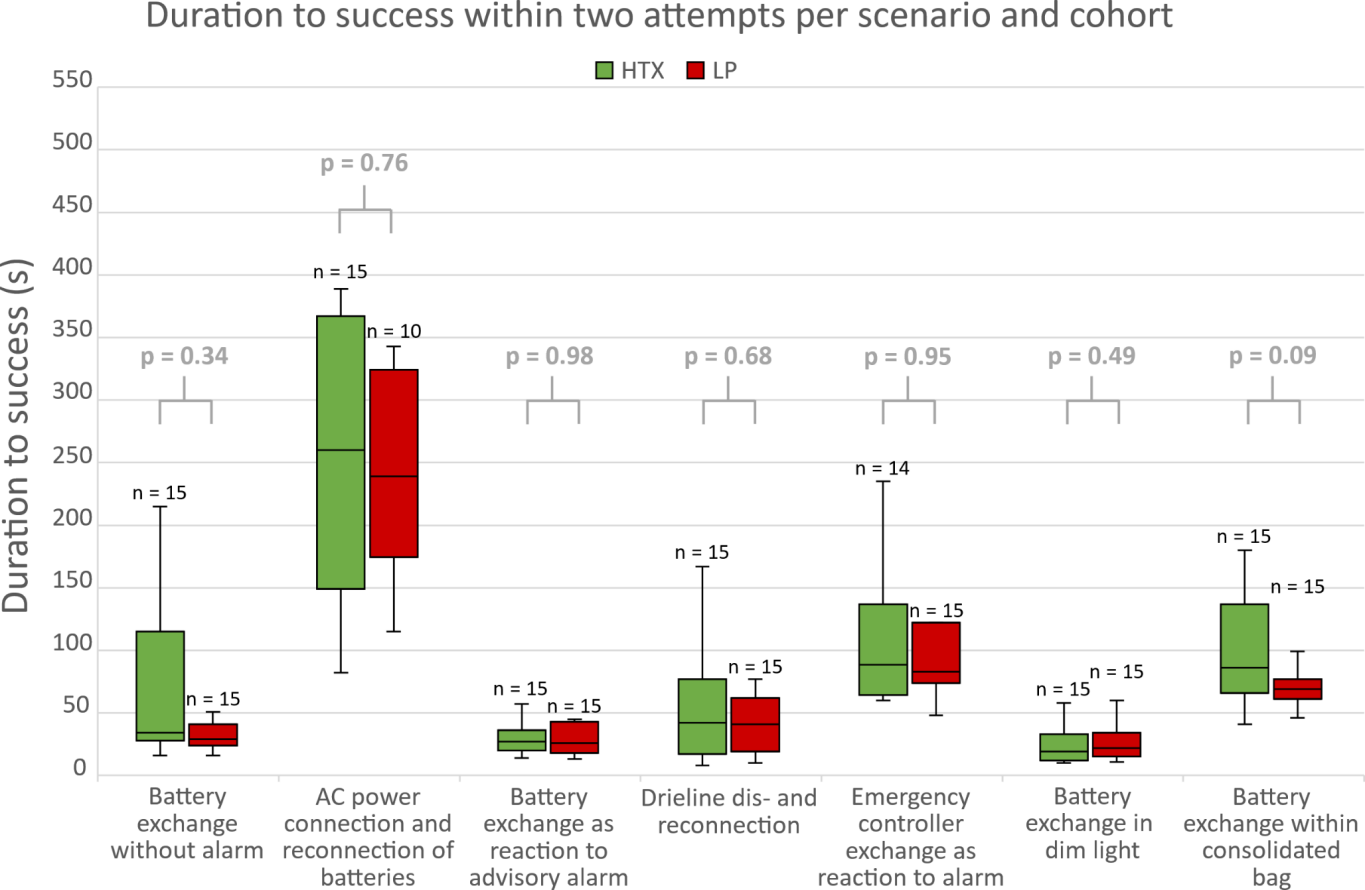
Duration to success in seconds per HeartMate 3 scenario and testing group (HTX: green, LP: red). HTX: heart transplantation; LP: laypersons

Overall, 82.4% (HTX: 82.9% vs. LP: 81.9%, p = 1.00) of the scenarios could be solved at first attempt, 17.8% at second attempt (16.2% vs. 13.3%, p = 0.69), and six scenarios (2.9%) could not be solved within two attempts (1.0% vs. 4.8%, p = 0.18). A summary of scenario performances is provided in Table 2, and duration to success (DTS) in Fig. 1.

Scenario specific, battery exchange without alarm was solved by 90.0% (HTX: 86.7% vs. LP: 93.3%, p = 1.00) of subjects at initial attempt, whereas 100% were able to succeed within two attempts, resulting in a duration to success (DTS) of 32.5 (18.8)s. Although LP solved it numerically faster (29.0 (17.0)s vs. HTX: 34.0 (87.0)s), no significant differences were found between cohorts (p = 0.34).

Change of power supply (AC power connection) was the most challenging of all 7 scenarios for both cohorts: only 26.7% (HTX: 33.3% vs. LP: 20.0%, p = 0.43) were able to complete the task within the three-minute time frame, including ten hazardous situations due to driveline disconnections (5 (33.3%) vs. 5 (33.3%), p = 1.00) resulting in unnecessary pump-off times from 2 to 118 s. Of 22 subjects who required a second attempt, significantly more LP failed (0 (0.0%) vs. 5 (41.7%), p = 0.04), resulting in a mean DTS of 251.2 ± 93.1s (255.9 ± 103.3s vs. 244.1 ± 80.3s, p = 0.76).

The driveline dis- and reconnection scenario was solved by 76.7% of the subjects at first attempt (HTX: 73.3% vs. LP: 80.0%, p = 1.00). Within two attempts, all subjects were able to succeed in a median DTS of 41.5 (44.0)s (42.0 (60.0)s vs. 41.0 (43.0)s, p = 0.68). Two subjects (1 vs. 1) were unable to reconnect the driveline following the disconnection, resulting in a permanent pump-stop.

The emergency controller exchange was successfully performed by 90.0% of the subjects (HTX: 86.7% vs. LP: 93.3%, p = 1.00) at initial attempt and by 6.7% (3.3% vs. 3.3%, p = 1.00) during the second attempt, with a median DTS of 83.0 (63.5)s (88.5 (72.5)s vs. 83.0 (48.0)s, p = 0.95) and pump-off time of 31.0 (61.3)s (30.0 (31.0)s vs. 32.0 (67.0)s, p = 0.51).

A high level of safety was achieved in the 4 battery exchange scenarios (success rate on the first attempt between 90% and 100%, Table 2). All former LVAD patients were able to succeed on first attempt during the battery exchange as reaction to advisory alarm, whereas two LP (13.3%) required a second attempt, with a median DTS of 26.5 (20.8)s (HTX: 27.0 (16.0)s vs. LP: 26.0 (25.0)s, p = 0.98). The battery exchanges in dim light and within the consolidated bag were solved by all subjects initially. Significantly decreased DTS during battery exchange (without alarm 32.5 (18.8)s vs. advisory alarm 26.5 (20.8)s and dim light 21.0 (19.0)s, p < 0.001), without significant differences between the groups (p > 0.34) indicate a high level of learnability and routine establishment. The battery exchange within the consolidated bag was more time consuming, especially for older subjects (r = 0.61, p < 0.001). Subjects with higher thumb-to-middle-finger-span performed the battery exchange within the bag faster (r=-0.56, p = 0.001). No further statistically significant correlations were found between scenario performances and demographics.

**Table 2:** Scenario performances of the overall study population and stratified by the two cohorts. Duration to success for subjects who succeeded within two attempts. * p-value comparing HTX vs. LP cohort. AC: alternating current; HTX: heart transplantation; LVAD: left ventricular assist device; LP: laypersons, IQR: interquartile range, STD: standard deviation

Scenario and performance variables Median (IQR) or mean ± STD or n (%)	Full cohort (n = 30)	HTX (n = 15)	LP (n = 15)	p-value*
**Battery exchange without alarm:**				
Success				
First attempt	27 (90.0%)	13 (86.7%)	14 (93.3%)	1.00
Second attempt	3 (10.0%)	2 (13.3%)	1 (6.7%)	1.00
No success	0 (0.0%)	0 (0.0%)	0 (0.0%)	
Duration to success	32.5 (18.8) s	34.0 (87.0) s	29.0 (17.0) s	0.34
Pump disconnection				
Yes	0 (0.0%)	0 (0.0%)	0 (0.0%)	1.00
Pump-off time	-	-	-	-
Any errors during first or second attempt	5 (13.3%)	4 (26.7%)	1 (6.7%)	0.10
**Change of power supply: battery operation to AC power and reconnection of batteries**				
Success				
First attempt	8 (26.7%)	5 (33.3%)	3 (20.0%)	0.43
Second attempt	17 (56.7%)	10 (66.7%)	7 (46.7%)	**0.04**
No success	5 (16.7%)	0 (0.0%)	5 (33.3%)	
Duration to success	251.16 ± 93.1 s	255.87 ± 103.3 s	244.10 ± 80.3 s	0.76
Pump disconnection				
Yes	10 (33.3%)	5 (33.3%)	5 (33.3%)	1.00
Pump-off time	11.0 (67.3) s	13.0 (101.5) s	6.0 (42.5)	0.25
Any errors during first or second attempt	24 (80.0%)	11 (73.3%)	13 (86.7%)	0.65
**Battery exchange as reaction to advisory alarm**				
Success				
First attempt	28 (93.3%)	15 (100%)	13 (86.7%)	0.54
Second attempt	2 (6.7%)	0 (0.0%)	2 (13.3%)	-
No success	0 (0.0%)	0 (0.0%)	0 (0.0%)	
Duration to success	26.5 (20.8) s	27.0 (16.0) s	26.0 (25.0) s	0.98
Pump disconnection				
Yes	0 (0.0%)	0 (0.0%)	0 (0.0%)	1.00
Pump-off time	-	-	-	-
Any errors during first or second attempt	6 (20.0%)	3 (20.0%)	3 (20.0%)	1.00
**Driveline dis- and reconnection**				
Success				
First attempt	23 (76.7%)	11 (73.3%)	12 (80.0%)	1.00
Second attempt	7 (23.3%)	4 (26.7%)	3 (20.0%)	1.00
No success	0 (0.0%)	0 (0.0%)	0 (0.0%)	
Duration to success	41.50 (44.0) s	42.0 (60.0) s	41.0 (43.0) s	0.68
Pump disconnection				
First attempt: Yes	25 (83.3%)	12 (80.0%)	13 (86.7%)	1.00
Second attempt: Yes	7 (100%)	4 (100%)	3 (100%)	1.00
Pump-off time	5.0 (6.3) s	5.0 (5.0) s	4.0 (8.0) s	0.51
Any errors during first or second attempt	10 (33.3%)	6 (40.0%)	4 (26.7%)	0.70
**Emergency controller exchange as reaction to hazard alarm**				
Success				
First attempt	27 (90.0%)	13 (86.7%)	14 (93.3%)	1.00
Second attempt	2 (6.7%)	1 (6.7%)	1 (6.7%)	1.00
No success	1 (3.3%)	1 (6.7%)	0 (0.0%)	
Duration to success	83.0 (63.5) s	88.50 (72.5) s	83.0 (48.0) s	0.95
Pump disconnection				
First attempt: Yes	29 (96.7%)	15 (100%)	14 (93.3%)	1.00
Second attempt: Yes	3 (100%)	2 (100%)	1 (100%)	1.00
Pump-off time	31.0 (61.3) s	30.0 (31.0) s	32.0 (67.0) s	0.32
Any errors during first or second attempt	20 (66.7%)	8 (53.3%)	12 (80.0%)	0.25
**Battery exchange in dim light**				
Success				
First attempt	30 (100%)	15 (100%)	15 (100%)	1.00
Second attempt	0 (0.0%)	0 (0.0%)	0 (0.0%)	1.00
No success	0 (0.0%)	0 (0.0%)	0 (0.0%)	
Duration to success	21.0 (19.0) s	19.0 (21.0) s	22.0 (19.0) s	0.49
Pump disconnection				
Yes	0 (0.0%)	0 (0.0%)	0 (0.0%)	1.00
Pump-off time	-	-	-	-
Any errors during first or second attempt	5 (16.7%)	3 (20.0%)	2 (13.3%)	1.00
**Battery exchange within consolidated bag**				
Success				
First attempt	30 (100%)	15 (100%)	15 (100%)	1.00
Second attempt	0 (0.0%)	0 (0.0%)	0 (0.0%)	1.00
No success	0 (0.0%)	0 (0.0%)	0 (0.0%)	
Duration to success	75.0 (45.0) s	86.0 (71.0) s	69.0 (16.0) s	0.09
Pump disconnection				
Yes	0 (0.0%)	0 (0.0%)	0 (0.0%)	1.00
Pump-off time	-	-	-	-
Any errors during first or second attempt	2 (6.7%)	2 (13.3%)	0 (0.0%)	0.48

### Eye Tracking

Of the 150 simulated everyday and emergency scenarios where ET was feasible, 35 recordings had to be excluded because of low gaze sample percentage and one recording due to a calibration error, resulting in 114 recordings eligible for ET analysis.

Percental fixation duration per AOI of HTX and LP during the battery exchange without alarm revealed that subjects with initial success primarily focused on the battery clips (HTX: 38.3 ± 8.0% vs. LP: 46.8 ± 15.4%, p = 0.14) and the initially connected batteries (25.9 ± 10.4% vs. 21.7 ± 12.8%, p = 0.42). Significant differences were observed in focusing irrelevant components (3.2 (6.8)% vs. 0.6 (1.4)%, p = 0.045).

Comparable results were obtained when changing batteries in response to the advisory alarm: participants in both groups focused their attention on the battery clips (HTX: 30.8 (33.9)% vs. LP: 31.3 (20.7)%, p = 0.82), but the HTX cohort paid significantly less attention to the connected batteries (10.9 ± 6.8% vs. 17.1 ± 5.4%, p = 0.028).

During the change of power supply, LP focused the cable connectors for a significantly higher proportion (HTX: 28.3 ± 4.3% vs. LP: 43.4 ± 1.8%, p = 0.002). Additionally, subjects of the LP cohort spent significantly less time on irrelevant components (9.5 ± 5.0% vs. 1.6 ± 0.3%, p = 0.04).

For the other scenarios, no significant differences in the percental fixation duration per AOI were observed between HTX and LP (dis- and reconnecting the driveline (p > 0.27), emergency controller exchange (p > 0.07)).

**Fig. 2:**
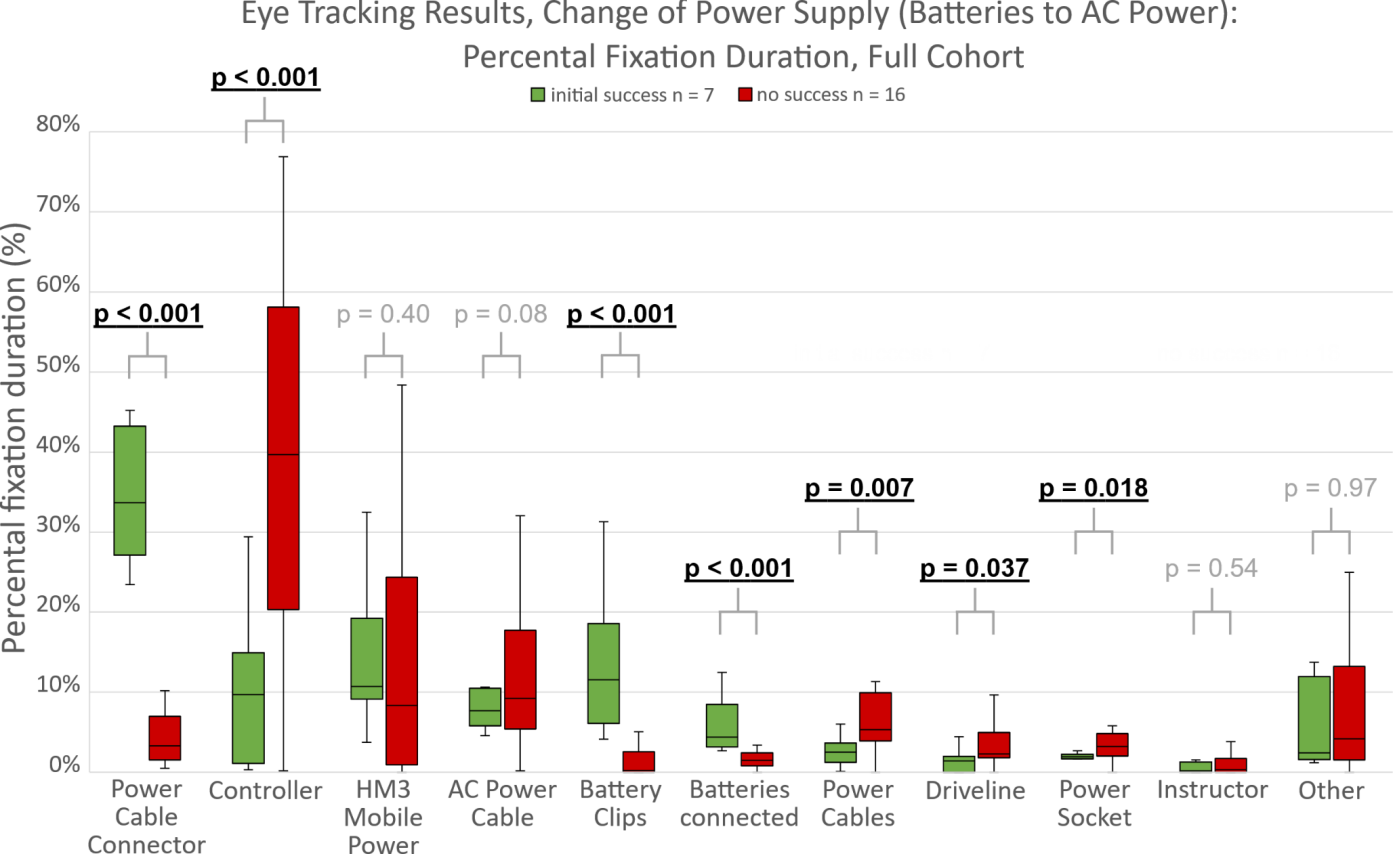
Percental fixation duration per area of interest and success (initial success: green, no initial success: red) during the AC power scenario

**Fig. 3:**
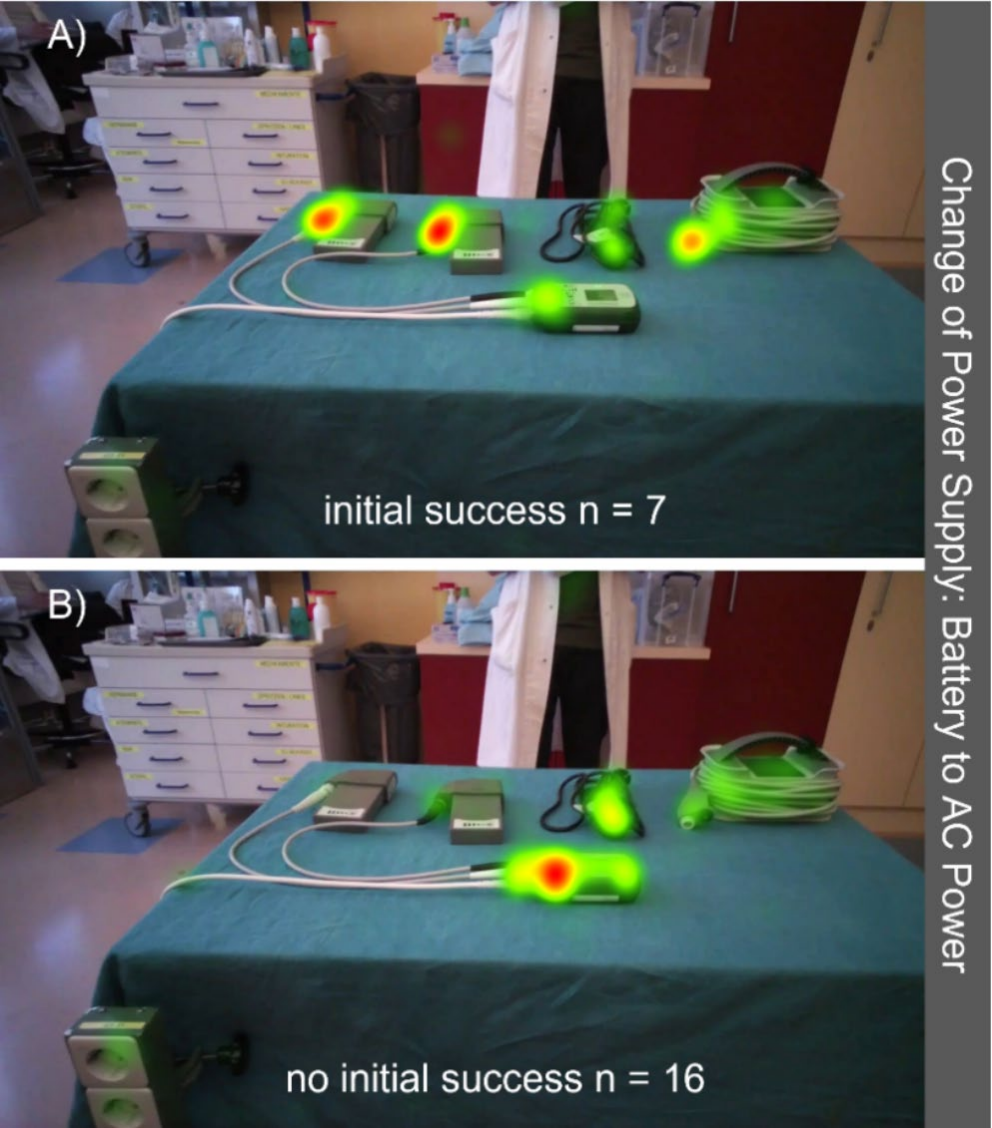
Heat maps generated based on relative fixation duration for AC power (alternating current) connection and reconnection of batteries. (A) Initially successful subjects. (B) Initially unsuccessful subjects

Stratified by success on first attempt (iSUC vs. iUNS), significant differences in gaze behavior were found during change of power supply (Figs. 2 and 3): successful subjects focused a significantly higher proportion on the battery clips (iSUC: 11.6 (12.5)% vs. iUNS: 0.2 (2.6)%, p < 0.001), cable connectors (33.7 (16.1)% vs. 3.3 (5.4)%, p < 0.001) and the connected batteries (4.4 (5.3)% vs. 1.5 (1.7)%, p < 0.001) and a significantly lower proportion on the controller (10.4 ± 9.9% vs. 40.0 ± 24.0%, p < 0.001), driveline (1.4 (2.0)% vs. 2.3 (3.2)%, p = 0.04), power cables (2.5 ± 1.9% vs. 6.0 ± 3.7%, p = 0.01) and the power socket (1.9 ± 0.6% vs. 3.6 ± 2.4%, p = 0.02).

In contrast, there were no significant differences in the dwell proportions between iSUC and iUNS participants for the emergency controller exchange as reaction to hazard alarm.

Additionally, HTX showed significantly higher changes in pupil diameter comparing introduction and task during the change of power supply (HTX: 6.0 ± 6.4% vs. LP: 0.4 ± 5.6%, p = 0.04) as well as during the emergency controller exchange (11.4 ± 8.1% vs. 4.4%±7.4%, p = 0.04). There were no other significant differences in pupil responses between the two cohorts or initial success.

### Survey

**Fig. 4:**
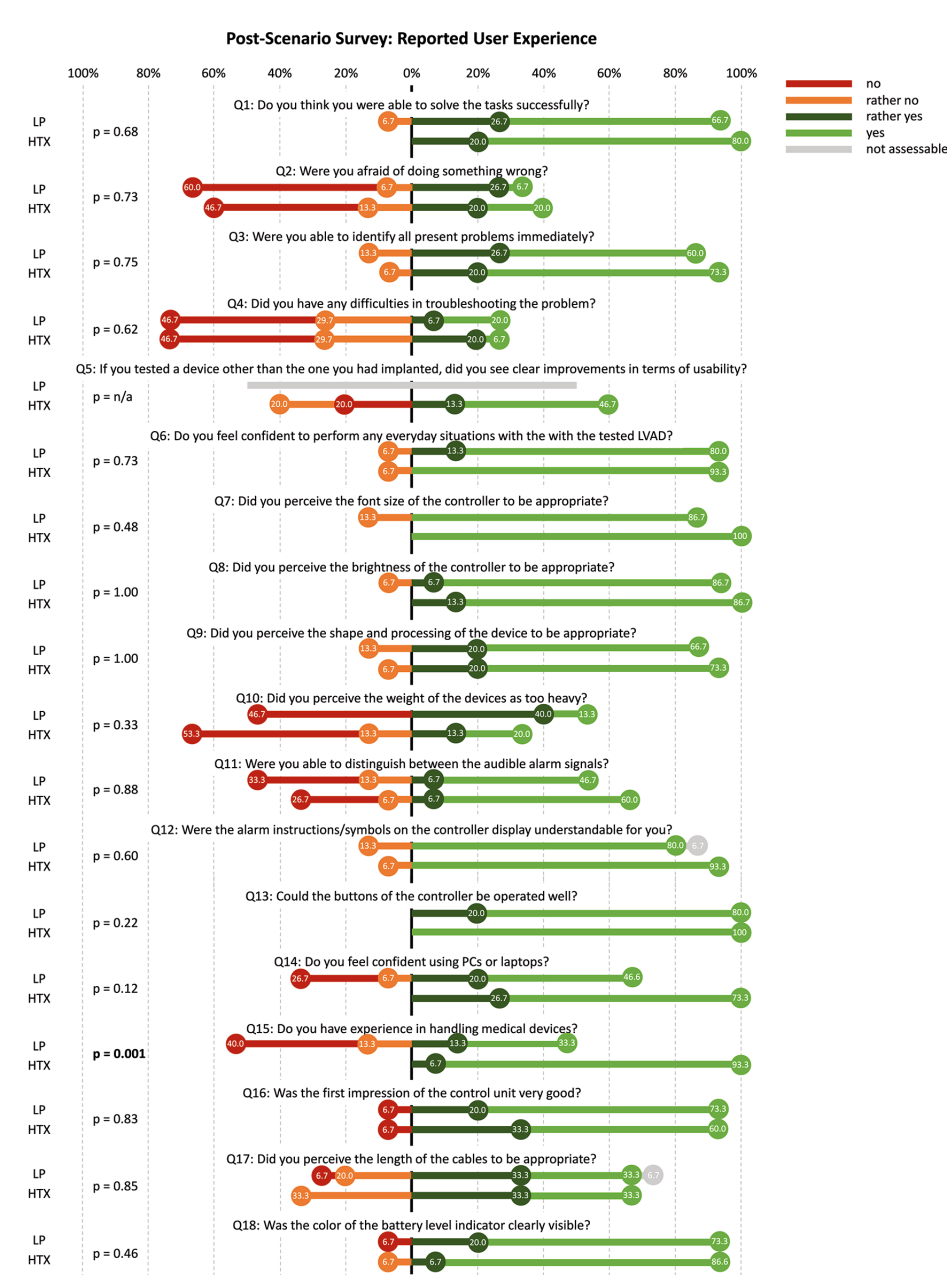
Results of 18-item Likert-Scale post scenario survey presented as percentage of cohort (former non-HM3 LVAD patients (HTX) vs. Laypersons (LP)). AC: alternating current, n/a: not applicable

The results of the post-scenario survey are summarized in Fig. 4. Although 63.3% (HTX: 60.0% vs. LP: 66.7%, p = 0.73) had no or rather no fear, 20.0% of former LVAD patients and 6.7% of LP were afraid of doing something wrong during the performed scenarios. Eight subjects (26.7% vs. 26.7%, p = 0.62) indicated that they rather or certainly had problems solving the scenarios. While 66.7% of subjects (60.0% vs. 73.3%, p = 0.83) had a good first impression of the controller, 40.0% of the HTX patients could not identify any improvement in usability compared to their previous LVAD. Additionally, 56.7% (66.7% vs. 46.7%, p = 0.33) agreed or rather agreed that the weight of the peripherals was too heavy. However, 86.7% of the subjects (93.3% vs. 80.0%, p = 0.73) would feel confident in handling everyday situations with the HM3 peripherals tested. Participants with fewer initially successful scenarios (≤ 5 vs. ≥6) reported more fear of doing something wrong (54.6% vs. 26.3%, p = 0.048), were less capable to identify the problems at hand (72.8% vs. 100%, p = 0.048), and less able to understand alarm instructions/symbols (72.7% vs. 94.7%, p = 0.041).

## Discussion

Despite improvements in pump technology and clinical outcomes [2–4, 28], limited usability and unintuitive handling remain as challenges for LVAD therapy [6–9, 12, 29]. Since non-pump malfunctions occur more frequently [13] and can be as critical as pump failure, it is essential to evaluate and optimize the usability of LVAD peripherals.

This study was the first to quantitatively and qualitatively assess the usability and intuitiveness of HM3 LVAD peripherals by using ET technology in simulated everyday and emergency scenarios further revealing unintuitive handling characteristics and design limitations. Although patients and their relatives are trained prior to hospital discharge, the most crucial steps in handling LVAD peripherals should be intuitive and manageable without previous experience, especially for first responders or EMS providers [10].

A major finding of this study was that success rates and DTS did not differ between LP and HTX (former non-HM3) patients, implicating that there was no apparent benefit from prior experience in handling other LVAD peripherals.

However, the high confidence in the four battery exchange scenarios (Table 2) indicate user-friendly and intuitive properties of the batteries and clips. Additionally, the significant decrease in DTS during the battery exchanges (comparing the battery exchange without alarm, as reaction to advisory alarm, and in dim light) can be considered as an indicator for high learnability and routine establishment.

In contrast, other clinically relevant findings of this study were (a) the high complexity of HM3 peripherals to change from battery to AC power and (b) the potentially hazardous driveline connector design:

Changing power supply (i.e., the screwing mechanism of the power cable connector) disclosed highest complexity, with a mean DTS of more than 4 min and only 26.7% successful first attempts, which may explain why 88.9% of HM3 patients sleep on batteries [12].

During the scenarios, four subjects were unable to reconnect the driveline, leading to a pump stop and associated hazards (Supplementary File 3). One participant inserted a misaligned driveline cable connector into the controller driveline connector, resulting in a fatal controller failure. Further, the unintuitive design of the driveline cable connector resulted in a median pump-off time > 30 s conducting the emergency controller exchange. During this period, patients would be solely dependent on their residual cardiac function, which may be too long to remain conscious, emphasizing that controller exchanges should not be performed by patients themselves without caregivers [30].

The use of ET technology, previously reported as not hindering or limiting participants in their performance [16, 31], provided the opportunity to understand the gaze behavior of successful and unsuccessful participants when changing power supply. Based on these results training materials could be modified and the findings should be considered in the next generation of peripherals to increase patient safety and satisfaction.

The simulated scenarios showed increased psychological burden and stress [22] in the HTX cohort, as measured by significantly higher changes in pupil diameter, particularly during emergency controller exchange, possibly due to knowledge of their former LVAD and the associated potential risks from maloperation. This was also confirmed in the post-scenario survey, where 20% of former LVAD patients were afraid of doing something wrong.

Finally, 40% of former LVAD patients could not identify clear improvements in usability compared with their previous device (HeartMate II and HVAD). Manufacturers of future LVAD peripherals should focus on a user-centered design [7, 12], taking into account all stakeholders involved, but especially the expertise of former LVAD patients.

This study has limitations that warrant discussion. First, due to the cross-sectional study design, the time since heart transplantation and thus since the last LVAD-equipment training varied from 6 months to 10 years in the HTX cohort. Second, a considerable number of ET recordings had to be excluded due to too low gaze sample percentage. Finally, to confirm the findings of this single center study an international multicenter should be conducted including greater heterogeneity of former LVAD systems, trained subjects and experts to benchmark against.

## Conclusion

This ET supported human factors simulation study provided insights into user experiences of untrained HTX patients and LP in handling HM3 peripherals. It highlighted highly complex and unintuitive handling properties during change of power supply and potentially hazardous characteristics of the driveline connector, providing guidance for future user-centered design of LVAD peripherals.

### Electronic Supplementary Material

Below is the link to the electronic supplementary material.

Supplementary Material 1

Supplementary Material 2

Supplementary Material 3
