# Supplementary material for: Human Factors Evaluation of HeartMate 3 Left Ventricular Assist Device Peripherals: An Eye Tracking Supported Simulation Study
Source: J Med Syst. 2023 May 3;47(1):58. doi: 10.1007/s10916-023-01950-3 (PMC10156833; doi:10.1007/s10916-023-01950-3)
Supplement: Supplementary file 1 — Supplementary Material 1 [file 10916_2023_1950_MOESM1_ESM.pdf]

Title: Human factors evaluation of HeartMate 3 left ventricular assist device peripherals: An eye tracking supported simulation study.

Journal name: Journal of Medical Systems

Authors: Gregor Widhalm[1], Theodor Abart[1], Moritz Noeske[1], Lisa Kumer[2], Katharina Ebenberger[1], Clemens Atteneder[1], Angelika Berger[2], Günther Laufer[1], Dominik Wiedemann[1], Daniel Zimpfer[1], Heinrich Schima[1,3,4], Michael Wagner[2], Thomas Schlöglhofer[1,3,4]

[1]Department of Cardiac Surgery, Medical University of Vienna, Vienna, Austria; [2]Division of Neonatology, Pediatric Intensive Care and Neuropediatrics, Department of Pediatrics, Comprehensive Center for Pediatrics, Medical University of Vienna, Vienna, Austria; [3]Ludwig Boltzmann Institute for Cardiovascular Research, Vienna, Austria; [4]Center for Medical Physics and Biomedical Engineering, Medical University of Vienna, Vienna, Austria

Email corresponding author: thomas.schloeglhofer@meduniwien.ac.at

Supplementary File 1: Provided components, patient introduction and initial setting per simulated scenario. (HM3: HeartMate 3, AC: alternating current)

Simulated scenario	Provided components	Subject Introduction	Initial Setting
Battery exchange without alarm	• Controller + 2 connected batteries<br>• Driveline to pump (mock loop)<br>• 2 spare batteries	"In front of you, you will find the controller including two batteries of a medical system, which is connected to the internal heart pump by means of the driveline, as well as two spare batteries.<br>Please now exchange both batteries of the system."	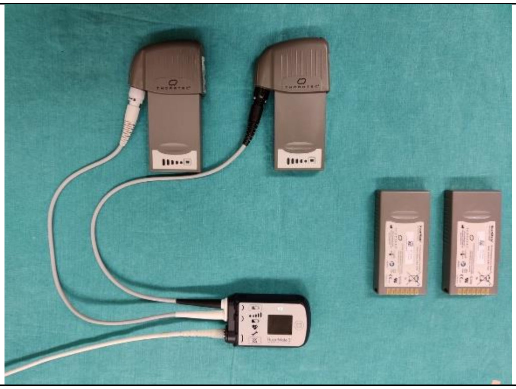

Simulated scenario	Provided components	Subject Introduction	Initial Setting
AC power connection and reconnection of batteries	• Controller + 2 connected batteries<br>• Driveline to pump (mock loop)<br>• HM3 Mobile Power Unit<br>• AC power cable	“In front of you, you will again find the medical device to be tested. In addition, a power socket can be found here (…).<br>Connect the controller to the power supply.<br>Subsequently, please disconnect the system from AC power supply and restore the original situation.”	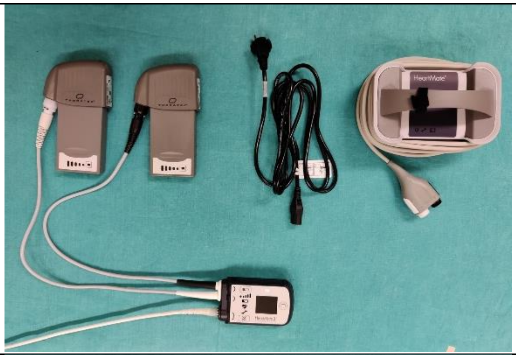
Battery exchange as reaction to alarm	• Controller + 2 connected batteries (low)<br>• Driveline to pump (mock loop)<br>• 2 spare batteries	“In front of you, you will again find the medical device to be tested.<br>You will see an error message on the display. In addition, you will perceive an acoustic alarm.<br>Please rectify the problem.”	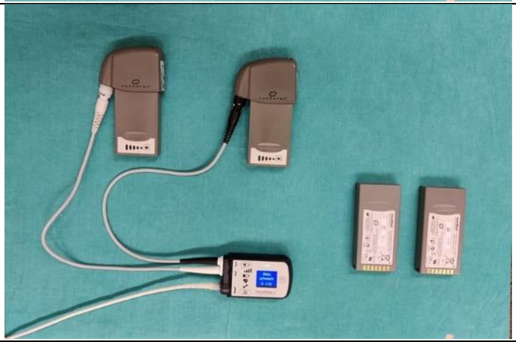
Driveline dis- and reconnection	• Controller + 2 connected batteries<br>• Driveline to pump (mock loop)	“In front of you, you will again find the medical device to be tested with a connection of the internal heart pump to the external control unit.<br>Please disconnect the control unit from the heart pump. This will trigger an alarm.<br>Re-establish the connection afterwards.”	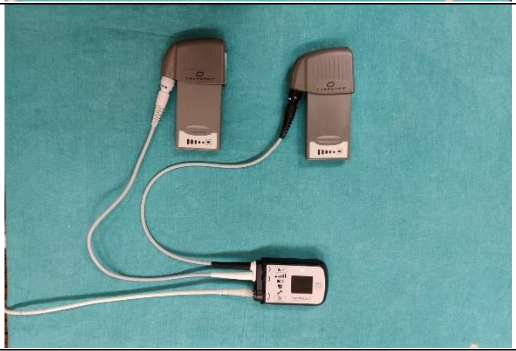

Simulated scenario	Provided components	Subject Introduction	Initial Setting
Emergency controller exchange as reaction to alarm	• Controller + 2 connected batteries<br>• Driveline to pump (mock loop)<br>• Spare controller	“In front of you, you will again find the medical device to be tested.<br>There is now a problem with the controller.<br>Please replace it with a new controller.”	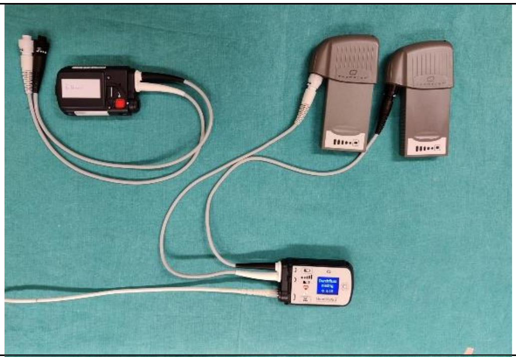
Battery exchange in dim light	• Controller + 2 connected batteries<br>• Driveline to pump (mock loop)<br>• 2 spare batteries	“The room is now darkened.<br>Please exchange both batteries of the system now.”	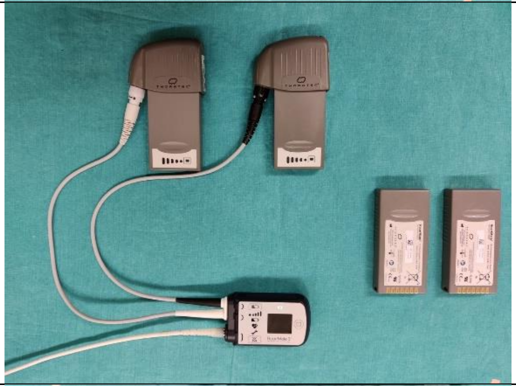
Battery exchange within consolidated bag	• Controller + 2 connected batteries (empty)<br>• Driveline to pump (sticked to the subject's abdomen)<br>• 2 spare batteries (empty	“During the next scenario, the components of the device are in a consolidated bag. We will put this consolidated bag on together. Please exchange both batteries while the controller remains in the bag provided.”	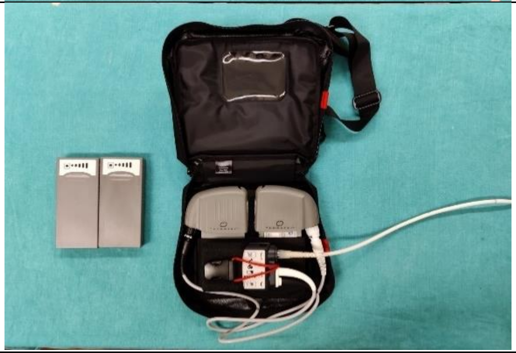
