# Supplementary material for: Human Factors Evaluation of HeartMate 3 Left Ventricular Assist Device Peripherals: An Eye Tracking Supported Simulation Study
Source: J Med Syst. 2023 May 3;47(1):58. doi: 10.1007/s10916-023-01950-3 (PMC10156833; doi:10.1007/s10916-023-01950-3)
Supplement: Supplementary file 2 — Supplementary Material 2 [file 10916_2023_1950_MOESM2_ESM.pdf]

Title: Human factors evaluation of HeartMate 3 left ventricular assist device peripherals: An eye tracking supported simulation study.

Journal name: Journal of Medical Systems

Authors: Gregor Widhalm[1], Theodor Abart[1], Moritz Noeske[1], Lisa Kumer[2], Katharina Ebenberger[1], Clemens Atteneder[1], Angelika Berger[2], Günther Laufer[1], Dominik Wiedemann[1], Daniel Zimpfer[1], Heinrich Schima[1,3,4], Michael Wagner[2], Thomas Schlöglhofer[1,3,4]

[1]Department of Cardiac Surgery, Medical University of Vienna, Vienna, Austria; [2]Division of Neonatology, Pediatric Intensive Care and Neuropediatrics, Department of Pediatrics, Comprehensive Center for Pediatrics, Medical University of Vienna, Vienna, Austria; [3]Ludwig Boltzmann Institute for Cardiovascular Research, Vienna, Austria; [4]Center for Medical Physics and Biomedical Engineering, Medical University of Vienna, Vienna, Austria

Email corresponding author: thomas.schloeglhofer@meduniwien.ac.at

Supplementary File 2: Defined areas of interest (AOIs) per provided HeartMate 3 component for the first five simulated scenarios.

**Battery exchange without alarm**
1. Controller
2. Batteries (initially connected)
3. Battery clips
4. Power cables
5. Power cable connector
6. Spare batteries
7. Driveline
8. Instructor
9. Other

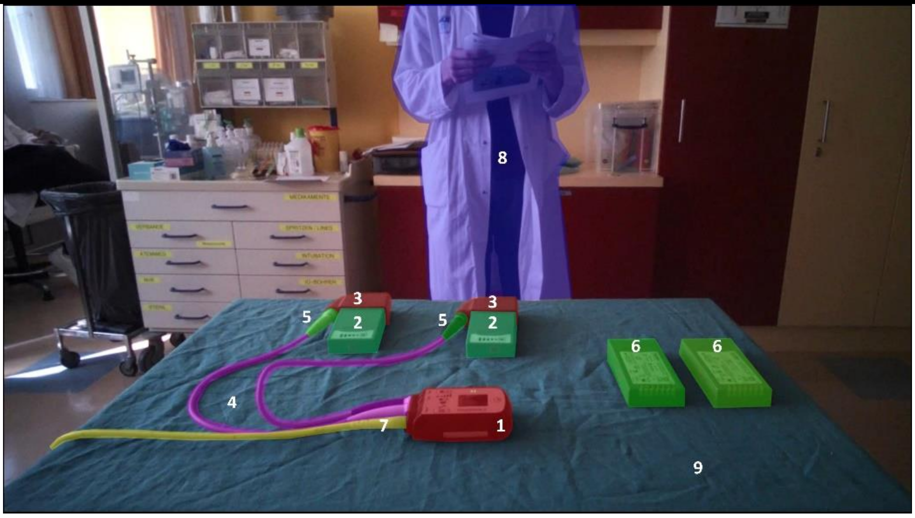

AC power connection and reconnection of batteries	
1. Controller	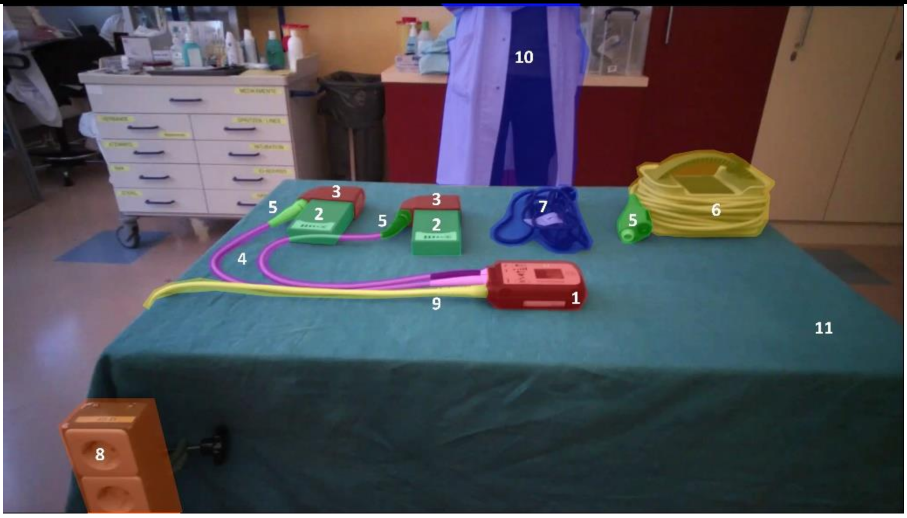
2. Batteries (initially connected)	
3. Battery clips	
4. Power cables	
5. Power cable connector	
6. HM3 Mobile Power Unit	
7. AC power cable	
8. Power socket	
9. Driveline	
10. Instructor	
11. Other	

Battery exchange as reaction to alarm	
1. Controller	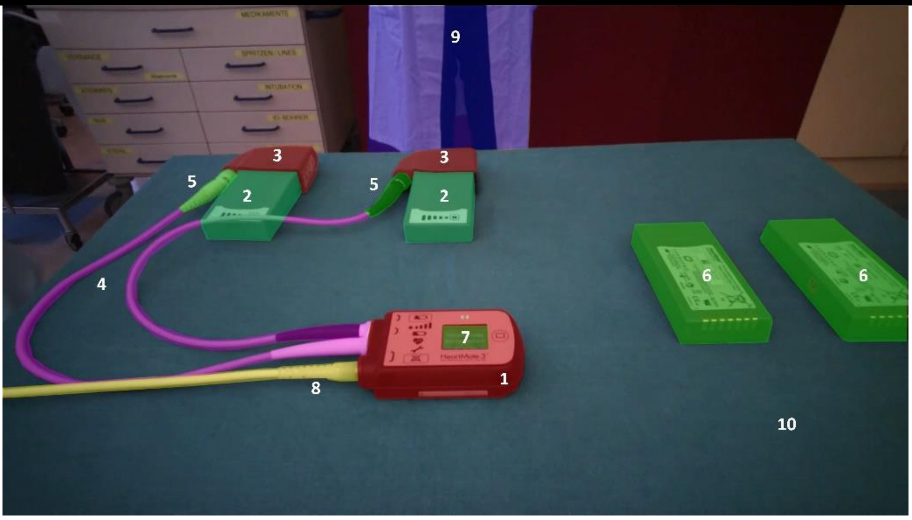
2. Batteries (initially connected)	
3. Battery clips	
4. Power cables	
5. Power cable connector	
6. Spare batteries	
7. Display (Warning)	
8. Driveline	
9. Instructor	
10. Other	

Driveline dis- and reconnection	
1. Controller	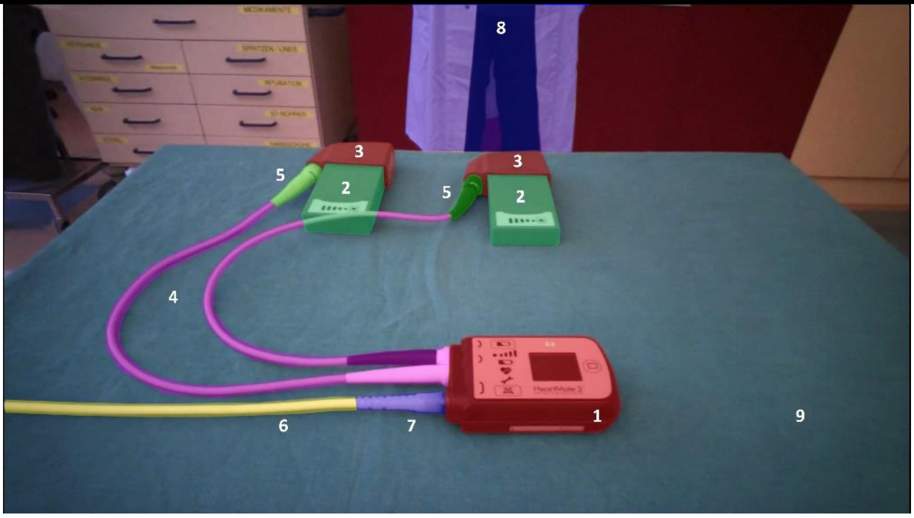
2. Batteries (initially connected)	
3. Battery clips	
4. Power cables	
5. Power cable connector	
6. Driveline	
7. Driveline connector	
8. Instructor	
9. Other	

Emergency controller exchange as reaction to alarm	
1. Controller	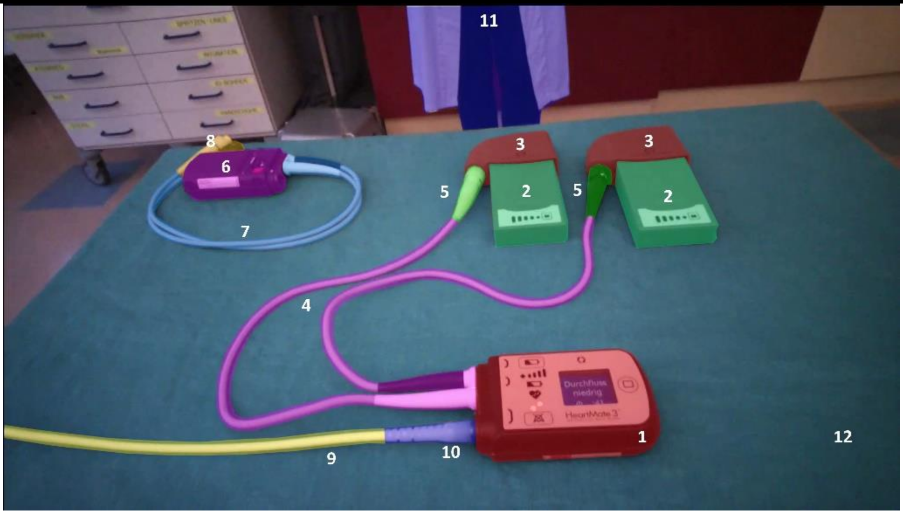
2. Batteries (initially connected)	
3. Battery clips	
4. Power cables	
5. Power cable connector	
6. Spare controller	
7. Spare controller power cables	
8. Spare power cable connector	
9. Driveline	
10. Driveline connector	
11. Instructor	
12. Other	
